# Farmers’ Exposure to Pesticides: Toxicity Types and Ways of Prevention

**DOI:** 10.3390/toxics4010001

**Published:** 2016-01-08

**Authors:** Christos A. Damalas, Spyridon D. Koutroubas

**Affiliations:** Department of Agricultural Development, Democritus University of Thrace, GR-682 00 Orestiada, Greece; skoutrou@agro.duth.gr

**Keywords:** Agricultural tasks, Direct spray contact, Drift, Occupational exposure

## Abstract

Synthetic pesticides are extensively used in agriculture to control harmful pests and prevent crop yield losses or product damage. Because of high biological activity and, in certain cases, long persistence in the environment, pesticides may cause undesirable effects to human health and to the environment. Farmers are routinely exposed to high levels of pesticides, usually much greater than those of consumers. Farmers’ exposure mainly occurs during the preparation and application of the pesticide spray solutions and during the cleaning-up of spraying equipment. Farmers who mix, load, and spray pesticides can be exposed to these chemicals due to spills and splashes, direct spray contact as a result of faulty or missing protective equipment, or even drift. However, farmers can be also exposed to pesticides even when performing activities not directly related to pesticide use. Farmers who perform manual labor in areas treated with pesticides can face major exposure from direct spray, drift from neighboring fields, or by contact with pesticide residues on the crop or soil. This kind of exposure is often underestimated. The dermal and inhalation routes of entry are typically the most common routes of farmers’ exposure to pesticides. Dermal exposure during usual pesticide handling takes place in body areas that remain uncovered by protective clothing, such as the face and the hands. Farmers’ exposure to pesticides can be reduced through less use of pesticides and through the correct use of the appropriate type of personal protective equipment in all stages of pesticide handling.

## 1. Introduction

The occurrence of harmful chemicals in the environment has become an issue of great debate in recent decades [1]. Pesticides and other foreign substances in food products and drinking water along with toxic pollutants in the air pose an immediate threat to human health, whereas other contaminants gradually build up in the environment and in the human body, causing disease long after first exposure [2]. It is also well known that many pesticides can accumulate in living species causing long-term and chronic effects, but there are difficulties in defining chronic exposure and disease outcomes, given the existence of a large series of variables of interest, such as lifestyle, occupation, diet preferences, and smoking, all of which must be taken into account to establish a disease-exposure relationship in the epidemiological investigation [3]. Chemicals play an important role in the efforts of countries to achieve economic growth and fulfill their development objectives [4], but, as much as they are vital for ensuring food security and economic growth, incorrect and indiscriminate use can be disastrous both for human health and the environment. In this context, chemicals can have a dual nature; they can be either beneficial or harmful, depending on numerous factors, such as the amounts to which exposure occurs [5]. Pesticides are common chemicals used to eliminate a great variety of unwelcome living organisms, particularly in agriculture. They are widely used in agriculture for the purposes of crop protection and in public health to control vector-borne infectious diseases. Because of high biological activity, and, in some cases, long persistence in the environment, pesticides may cause harmful effects to human health and to the environment [6]. Improper handling may result in severe acute poisonings; in some cases, adverse health effects may also result from long-term, low-level exposures [7]. As a result of widespread diffusion of pesticides, a great part of the population may be exposed to pesticides due to occupation. Several groups of people, characterized by quite different patterns and degree of exposure, face the risk of adverse effects. Occupational exposure typically occurs in workers involved in the manufacture of pesticides and among specific users in public health (e.g., exterminators of house pests). In the agricultural sector, exposure to pesticides typically occurs among farmers and professional applicators of pesticides [7,8,9,10]. Regarding the general population, individuals may be exposed to pesticide residues in food and drinking water on a daily basis or to pesticide drift in residential areas close to spraying areas [11].

The greatest amount (about 75%) of pesticide use in the USA occurs in agriculture [12]. In Europe, despite international efforts to promote the sustainable use of pesticides in agriculture and an actual reduction in use in several countries, overall pesticide use did not decline substantially in the WHO European Region during the period of 1990s [13]. In developing nations, many old, non-patented, more toxic, environmentally persistent, and inexpensive types of chemicals are used extensively, creating significant acute health problems and also local environmental contamination [14,15,16]. As a result, farmers and farm workers face greater risk of exposure to pesticides than typical non-agricultural workers, comprising a major group of workers that are consistently exposed to pesticides. The type of pesticides, the frequency of use, and the application method are usually different according to the farm type and the specific crops grown. Pesticide use is a seasonal, but intermittent task, covering only one of a wide range of tasks undertaken by farmers. Consequently, the frequency and the total time of exposure for most farmers are generally lower than for pesticide applicators in other industries. Dedicated pesticide applicators in the agricultural sector are exposed to pesticides more frequently than farm operators, even though they may have fewer years of pesticide use. Many studies of pesticide exposure have focused mainly on farm workers who are certified pesticide applicators. However, there is enough evidence that pesticide exposure may not be universal among farm workers and that a great proportion of farmers may not be exposed to pesticides directly.

Recently, the potential of certain pesticides to act as endocrine disrupting chemicals (EDCs) has been assessed [17]. These pesticides include certain organometallic compounds and many older organochlorine compounds that are also toxic and persistent. The latter were banned from use in many countries in the 1970s and 1980s, so exposures to these compounds have been decreasing ever since. However, these chemicals are highly persistent and small amounts are still present in the environment. Due to the uncertainty surrounding how much of a chemical is needed to have an impact, further research is required to allow determination of the best management approach. Other pesticides such as organophosphates, carbamates, triazines, and pyrethroids that are less persistent and less toxic than the organochlorines were used to replace them, but many are now confirmed or suspected to act as endocrine disrupting chemicals [18]. Some endocrine disrupting pesticides show toxic effects that decrease as the dose decreases, until, at very low doses (often as low as parts per billion or even parts per trillion), their effects increase [19].

Regular summaries of the relevant literature usually are both advantageous and necessary for scientists, given the large volume of papers published every year. They can lead to new synthetic insights and are often widely read. The aim of this editorial was to summarize basic knowledge on farmers’ exposure to pesticides. The editorial is primarily descriptive and is not intended to be a comprehensive review on pesticide exposure; such reviews are referenced when they are available for a specific related topic.

## 2. Pesticide Toxicity and Risk (Hazard)

**Figure 1:**
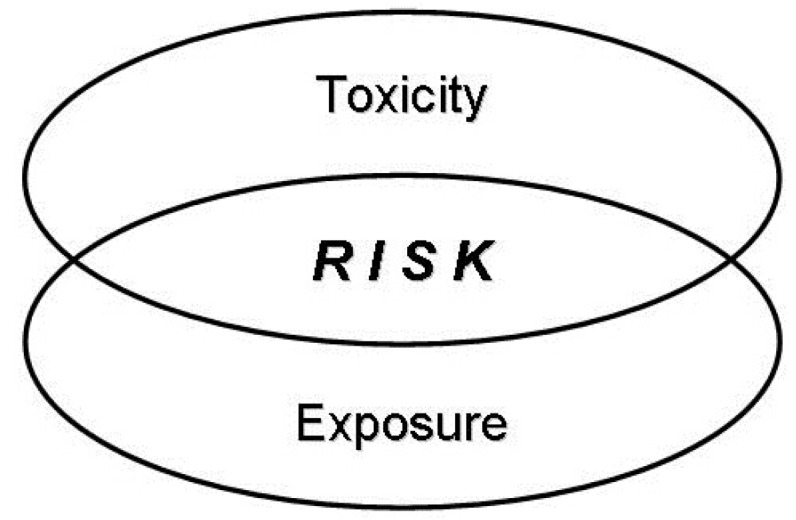
Risk is a combination of toxicity and exposure.

In terms of pesticide safety, there is a difference between the words “toxicity” and “risk”. Toxicity refers to inherent poisonous ability of a material [20]. The toxicity of a material is evaluated in toxicology laboratories and is expressed in quantitative terms, such as LD_50_ or LC_50_ (lethal dose or concentration 50%, *i.e.*, the dose or concentration at which a material will kill 50% of a reference organism). The risk (or hazard) depends not only on the toxicity of a material, but also on the possibility of exposure when used. In simple terms, toxicity is the capacity of a substance to produce illness or even death, whereas risk (hazard) is a combination of toxicity and exposure (Figure 1). Therefore, the risk (hazard) from a specific pesticide depends on the toxicity of the specific product used and the amount and form of exposure experienced.

Information about both toxicity and exposure is required to determine risk (hazard). Normally, the potential for adverse effects to humans from highly toxic pesticides is greater than that from pesticides that are less toxic [20]. However, other factors, such as the concentration of the pesticide in a formulation, the length of exposure to the pesticide, and the route of entry into the human body, are of major importance in the potential for poisoning [21]. Evidently, a pesticide applicator can have limited control over the toxicity of a pesticide, but significant control over risks that are associated with the use of this pesticide can be expected. For example, a container of a highly toxic pesticide that is sealed presents little risk before the seal is broken. Even in the case when the container is opened, the risk remains small, unless the end user is not wearing protective gear. However, if the container is cracked or leaking, or if proper protective gear is not used, the risk can be serious.

Pesticides may harm humans via poisoning or injuries. Poisoning is caused by pesticides that affect organs or systems inside the body, whereas injuries are usually caused by pesticides that are external irritants. Some pesticides are highly toxic to humans; only small amounts can cause highly harmful effects. Other active ingredients are less toxic, but overexposure to them also can be detrimental. Toxic effects by pesticide exposure can range from mild symptoms, like minor skin irritation or other allergic symptoms, to more severe symptoms, like strong headache, dizziness, or nausea. Some pesticides, e.g., the organophosphates, can cause severe symptoms, like convulsions, coma, and possibly even death. Pesticide toxicity in humans can be categorized by the nature of exposure, the route through which exposure occurs, or the body system affected. As a general rule, any poison is more toxic if ingested than if inhaled and more toxic if inhaled than if absorbed by the skin (dermal exposure). Some toxic effects by pesticides are temporary, given that they are quickly reversible and do not cause severe or permanent damage. Certain pesticides may cause reversible damage, but full recovery may take long periods of time. Still other poisons may have irreversible effects, although the exposure is not fatal.

## 3. Classification of Toxicity by Type of Exposure

In human exposure situations, toxicity by pesticides may be divided into three main types, based on the exposure time to the pesticide and how rapidly the toxic symptoms develop [22]. Thus, workplace or environmental exposure may be described as acute, sub-chronic, and chronic (Table 1). When a farmer is exposed to a single dose of a pesticide, the incidence is referred to as acute exposure and the effect is called acute toxicity. Acute toxicity refers to how poisonous a pesticide is to an organism after a single short-term exposure (Table 1). If the exposure is through contact with skin, it would be regarded as an acute dermal exposure event and the toxicity is called acute dermal toxicity. Similarly, acute oral exposure refers to a single dose of a pesticide taken by mouth, and acute inhalation exposure refers to a single dose inhaled. The acute toxicity is used to describe toxic effects which typically appear immediately or within a day (24 h) of exposure. An active ingredient with a high acute toxicity can be lethal even when a very small amount is absorbed. The acute toxicity of a pesticide is used for the warning statements on the product label.

**Table 1:** Types of toxicity based on the extent of exposure to a pesticide [23].

Type	Definition
Acute toxicity	Occurring from a single incident of exposure (single short-term exposure).
Subchronic toxicity	Occurring from repeated incidents of exposure over several weeks or months (intermediate exposure, normally less than the lifetime of the exposed organism).
Chronic toxicity	Occurring from repeated incidents of exposure for many months or years (repeated long-term exposure, sometimes lasting for the entire life of the exposed organism).

The signal words displayed on the product label (Table 2) are selected on the basis of the acute toxicity of the pesticide product. Subchronic toxicity is the ability of a chemical compound to cause toxic health effects for over a year, but less than the lifetime of the exposed organism. In cases of continuous exposure to a pesticide occurring repeatedly by an individual, the incidence is referred to as chronic exposure. This effect can be reported as chronic dermal, chronic oral or chronic inhalation toxicity.

**Table 2:** Types of acute toxicity measures and warnings [23].

Categories	Signal Word	Oral mg/kg	Dermal mg/kg	Inhalation mg/L
I—Highly toxic	POISON	0 to 50	0 to 200	0 to 0.2
II—Moderately toxic	WARNING	50 to 500	200 to 2,000	0.2 to 2.0
III—Slightly toxic	CAUTION	500 to 5,000	2,000 to 20,000	2.0 to 20
IV—Relatively non-toxic	CAUTION	5,000+	20,000+	20+

Chronic toxicity is the ability of a pesticide to cause adverse health effects over an extended period, usually after repeated or continuous exposure, which may last for the entire life of the exposed organism. This type of pesticide toxicity is of concern not only to the general public, but also to those working directly with pesticides, given the potential exposure to pesticides found on/in commodities, water, and the air. It is measured in experimental conditions usually after a period of three months of either continuous or occasional exposure. A pesticide that has high acute toxicity does not always have high chronic toxicity. Nor will a pesticide with low acute toxicity necessarily have low chronic toxicity. For many active ingredients, the toxic effects from single acute exposure are quite different from those produced by chronic exposure. The small amount of a pesticide that is absorbed from a single exposure is rather insufficient to cause illness, but absorption of the same small amount every day continuously can cause serious chronic illness or even death. The effects of acute toxicity and chronic toxicity are dose-dependent; the greater the dose, the greater the effect. In characterizing the toxicity of a pesticide, it is evident that information is needed for the single-dose (acute) and the long-term (chronic) effects, including also information for exposure of intermediate duration. For example, delayed toxicity may occur many years after exposure to a chemical. A major differentiation is that a delayed toxic reaction is not identical to the chronic adverse effects. In contrast to chronic exposure, which typically refers to continuous exposure to low levels of a toxicant, delayed toxicity can be a result of a single dose or a brief exposure event, producing a permanent effect [24]. Consequently, dose, duration, and exposure issues for delayed toxicity are not comparable to those for chronic exposure. In fact, epidemiological studies are important to the detection of further occurrences of delayed toxicity.

## 4. Classification of Toxicity by Route of Entry

**Figure 2:**
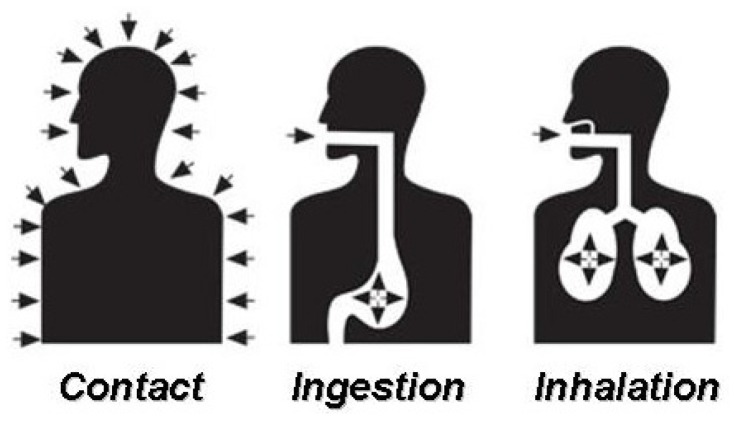
Main routes of pesticide entry in the human organism [8].

Pesticides can enter the human body by three common ways: through the skin (contact), the mouth (ingestion), and the lungs (inhalation) (Figure 2). The state of the chemical, *i.e.*, solid, liquid, or gas, affects the chances of pesticide penetration into the body [25]. Liquid or gas products can get into the body through all three routes of entry, whereas solids tend to have a lower chance of entry through the lungs. However, if solid particles of the pesticide are small enough or if they remain on the skin long enough, penetration into the body can take place in the same ways as those of liquids or gases. The most common pathway for pesticide poisoning among common users is absorption through the skin [26]. Dermal absorption may occur as a result of splashes and spills when handling (mixing, loading or disposing of) pesticides. To a minor degree, dermal absorption may occur from exposure to great load of residues. The degree of hazard by dermal absorption depends on the toxicity of the pesticide to the skin, the duration of the exposure, the pesticide formulation, and the body part contaminated [27]. Powders, dusts, and granular pesticides are not absorbed so easily through the skin and other body tissues as are the liquid formulations. On the other hand, liquid pesticides containing solvents (e.g., organic solvents) and oil based pesticides usually are absorbed more quickly than dry pesticides. For example, the emulsifiable concentrates, containing a great percentage of the toxic substance in a relatively small amount of solvent, are readily absorbed by the skin. Certain body areas are more prone to absorption of pesticides than other areas.

Pesticides entering the body through the mouth (oral exposure or also called ingestion) may cause serious illness, severe injury, or sometimes even death [21]. These products may be consumed inadvertently (*i.e.*, accidental oral exposure) or may be consumed intentionally by individuals who intend on personal harm (*i.e.*, deliberate self-poisoning). Oral exposure can also occur when hands are not properly washed before eating or smoking. Furthermore, pesticides may be swallowed by mistake, when improperly stored in food containers. Materials that are ingested can be absorbed along the gastrointestinal tract, with the small intestine being reported to be the major absorption site. Once absorbed, they find their way into the blood stream, through which they are capable of readily distributing throughout the entire body. Frequent cases of accidental oral exposure, probably the most frequent, are those in which pesticides have been moved from their original labeled container to an unlabeled bottle or food container. There are many cases where people have been poisoned by drinking pesticides or water stored in pesticide-contaminated bottles. Deaths from pesticide poisoning contribute significantly to patterns of suicide, particularly in rural areas of developing nations.

Pesticides entering the body through inhalation can cause serious damage to the nose, the throat, and the lung tissues. The rapid absorption of pesticides through this specific route increases the risk of respiratory exposure. The greatest potential for poisoning via respiratory exposure is with vapors and extremely fine particles of the spray solution. Pesticide exposure is usually low when dilute sprays are applied with common conventional spraying equipment because larger droplet sizes are produced. By contrast, when low volume equipment is utilized to apply concentrated material, the potential for an event of respiratory exposure is increased because smaller droplets are produced. Pesticide application in confined areas (e.g., greenhouses) also contributes to high potential for exposure through inhalation. Respirators and gas masks can provide protection from respiratory exposure. Eyes are particularly sensitive to absorption, and therefore any contact of pesticides with the eye presents an immediate threat of injury, blindness, or sometimes even death. Eye protection is always a prerequisite when measuring or mixing concentrated and toxic pesticides. Proper protection of the eyes should also be used when there is a chance of exposure to the diluted spray or dusts that may drift into the eyes. Pesticides in a granular formulation may also present a high risk to the eyes due to the size and weight of the individual particles. If applied with power equipment, particles may bounce off vegetation and cause significant eye injury as well as poisoning to an applicator if struck in sensitive body areas (*i.e.*, the eyes). Therefore, protective goggles should be used whenever there is a possibility of pesticides coming into contact with the eyes.

## 5. Pesticide-Related Work Tasks

Pesticide use is typically associated with three basic stages: (i) mixing and loading the pesticide product, (ii) application of the spray solution, and (iii) clean-up of the spraying equipment. Mixing and loading are the tasks associated with the greatest intensity of pesticide exposure, given that during this phase farmers are exposed to the concentrated product and, therefore, often face high exposure events (e.g., spills). However, the total exposure during pesticide application may exceed that incurred during mixing and loading, given that pesticide application typically takes more time than the tasks of mixing and loading. Pesticide drift is also a permanent hazard in pesticide use, because it exists even in the most careful applications, and therefore, can increase the possibility of detrimental effects of pesticide use on the users and the environment [28]. There is also evidence that cleaning the equipment after spraying may also be an important source of exposure. The level of pesticide exposure to the operator depends on the type of spraying equipment used. Hand spraying with wide-area spray nozzles (when large areas need to be treated) is associated with greater exposure to the operator than narrowly focused spray nozzles. When pesticides are applied with tractors, the application equipment is mounted directly on the tractor and is associated with a higher degree of operator exposure than when the spray equipment is attached to a trailer. Pesticide deposition on different parts of the operator’s body may vary largely due to differences in individual work habits. Several studies on the contamination of the body in pesticide applicators showed that the hands and the forearms suffer the greatest pesticide contamination during preparation and application of pesticides. However, other body parts such as the thighs, the forearms, the chest, and the back may also be subject to significant contamination.

Clean-up of the spraying equipment is an important task in the use of pesticides. The time given to the task of cleaning may occupy a considerable part of the basic stages of pesticide handling [29,30]. Despite considerable variation among farm workers, equipment cleaning has been found to contribute greatly to workers’ daily dermal exposure [29]. Unexpected events, such as spills and splashes, are also a major source of dermal contamination for pesticide applicators, and often the exposure from these events can result in significant acute and long-term health effects [30]. Spills and splashes usually occur during mixing or loading and application, but may also appear in the stage of equipment clean-up [29]. Farmers (or farm workers) who make the spray solutions and apply pesticides have been at the center of attention of most research thus far, but often farmers re-entering the sprayed fields may also face pesticide exposure, sometimes to significant levels [31,32]. It is not surprising that re-entry farm workers may face even greater exposure than pesticide applicators, possibly because safety training and the use of PPE are usually less, and the duration of exposure may be greater than that of the applicators [31,32,33]. Exposure by re-entry in the sprayed fields may become a serious problem if farm workers re-enter the treated fields soon after pesticide application [34]. Spray drift from neighboring fields and overexposure events of this kind, each involving groups of workers, have been documented as inadvertent events of farmers’ exposure to pesticides [35].

## 6. Reducing Exposure to Pesticides

### 6.1. Alternative Cropping Systems Less Dependent on Pesticides

To achieve the desirable goal of minimum exposure to pesticides, it is essential to shift towards alternative cropping systems that are less dependent on pesticides. This can be realized by focusing more on ecological approaches of crop protection based on available ecological knowledge. The use of advanced ecological knowledge by agronomists is fairly recent. The purposes of this approach are to increase the abilities of agricultural systems to induce the natural processes of pest regulation and to contribute to the improvement of the agricultural production. Sustainable systems of pest, disease, and weed management should include three basic elements: prevention, decision making, and control [36]. Prevention can be optimized by maximizing the use of natural processes in the cropping system, suppressing the harmful organisms by promoting the development of antagonists, optimizing the diversity of the system, and stimulating the recycling of internal resources [37]. Instruments to achieve that may include: (i) farm hygiene with the important element of the use of clean seed or planting material and maintaining temporal and spatial separation between crops of the same species (e.g., control of volunteers), (ii) synergistic and antagonistic effects occurring in a cropping system, e.g., the suppression of diseases and pests by a designed system of non-chemical preventive methods, including the cultivation of catch crops and the use of soil amendments to enhance populations of antagonists, (iii) cultural practices that support ecological processes, such as delayed planting to reduce weed growth or even prevent seed set, removal of crop residues or plant debris, management of soil organic matter, and soil tillage strategies, (iv) optimization of other inputs such that a crop can grow in a healthy condition that will assist in withstanding attacks of pathogens or that will increase the damage threshold, (v) breeding for tolerance, e.g., by selecting for specific plant types that are more competitive against weeds or resistant to diseases, e.g., against blights.

### 6.2. Use of Personal Protective Equipment (PPE)

Various types of personal protective equipment (PPE) can be used in pesticide handling to limit dermal exposure. Gloves, boots, hats, long sleeve shirts, and chemical-resistant coveralls are among the most common types of PPE. The toxicity of the pesticide used, the circumstances of exposure, and the worker’s personal preferences ultimately affect the type of PPE used among farmers. The use of gloves and boots are the minimum PPE for most pesticide products. As a general rule, highly toxic pesticides require the use of multiple types of PPE for reducing exposure. Different types of PPE provide complementary levels of personal protection against dermal exposure. Wearing gloves was found to be the most effective protection method against pesticide exposure occurring among Danish greenhouse workers, and reduced dermal exposure among US citrus farmers by 27%. In the latter case, dermal exposure among US citrus farmers was reduced by 65% when both gloves and coveralls were used.

The PPE provides protection that may vary according to the protective features of each type of PPE itself, the way in which the pesticide is applied, and the level of correct fitting and maintenance by the farmers. Common protective clothing provides protection against exposure according to fabric type, including thickness and weight. Garments of both barrier and non-barrier fabrics were found to decrease dermal exposure [38]; however, greater protection was found by waterproof polypropylene fabrics compared with cotton garments [39]. Penetration through cotton clothing ranged from 11.2% to 26.8%, whereas in the case of synthetic material, penetration was found to be less than 2.4% [40]. However, little difference was found between synthetic and woven fabrics in a study of US citrus farmers [41]. The effectiveness of PPE in terms of pesticide penetration through clothing has been reported to be affected by the application method [42,43,44]; however, results concerning this issue have been inconsistent. For example, while low-pressure backpack spraying was associated with greater pesticide penetration through the clothing than high-pressure spraying [45], according to other research [40], a low-pressure backpack application resulted in lower penetration than high-pressure hand lance spraying.

An important determinant of the effectiveness of any PPE, which often gets unnoticed, is the way in which each PPE is actually used. Often, farmers’ movements during pesticide application that promote the relocation and further spread of dust or liquids via PPE fabric along with farmers’ sweating, particularly in hot environments, also affect penetration resistance of the PPE fabric [41]. Parts of a polyethylene coverall showed greater penetration, taking into account that the movement of farmers is likely to create friction [46]. Obviously, the protective ability of any PPE depends on proper use. For example, farmers who often roll sleeves up or take gloves off in the middle of pesticide handling are at increased risk of dermal exposure [40]. Personal protection can be low because the PPE is unsuitable, incorrectly fitted, not properly maintained, and improperly used. Thus, the theoretically maximum levels of protection are seldom achieved with routinely use of PPE, and the actual level of personal protection is difficult to assess.

Pesticides will remain a tool for modern agriculture, so it is important to design strategies that will reduce pesticide impact [47]. This can be achieved with minimum use of pesticides using accurate diagnosis and advanced knowledge of pest problems, optimized timing of interventions for maximum long-term efficiency, selection of a pesticide product with minimum impact on non-target organisms and the operator, and improved application of the selected product for maximum dose transfer to the biological target [48]. The overall optimization of the procedure of pesticide handling, strictly following the regulations and taking into account the public concerns with reference to pesticide residues in food and drinking water are essential. To this end, whenever pesticides are used, operative and well-maintained spraying equipment and the necessary precautions at all stages of pesticide handling are essential for reducing farmers’ exposure to pesticides.
